# The Role of PEMFs on Bone Healing: An In Vitro Study

**DOI:** 10.3390/ijms232214298

**Published:** 2022-11-18

**Authors:** Laura Caliogna, Valentina Bina, Alice Maria Brancato, Giulia Gastaldi, Salvatore Annunziata, Mario Mosconi, Federico Alberto Grassi, Francesco Benazzo, Gianluigi Pasta

**Affiliations:** 1 Orthopedics and Traumatology Clinic, IRCCS Policlinico San Matteo Foundation, 27100 Pavia, Italy; 2 Department of Molecular Medicine, University of Pavia, 27100 Pavia, Italy; 3 Centre for Health Technologies, University of Pavia, 27100 Pavia, Italy; 4 Department of Clinical, Surgical, Diagnostic and Pediatric Sciences, University of Pavia, 27100 Pavia, Italy; 5 Sezione di Chirurgia Protesica ad Indirizzo Robotico-Unità di Traumatologia dello Sport, U.O. Ortopedia e Traumatologia Fondazione Poliambulanza, 25124 Brescia, Italy

**Keywords:** pulsed electromagnetic fields (PEMFs), human adipose mesenchymal stem cells (hASCs), human osteoblasts (hOBs), osteogenic differentiation, fracture repair, fracture healing, bone regeneration

## Abstract

Bone responses to pulsed electromagnetic fields (PEMFs) have been extensively studied by using devices that expose bone cells to PEMFs to stimulate extracellular matrix (ECM) synthesis for bone and cartilage repair. The aim of this work was to highlight in which bone healing phase PEMFs exert their action. Specifically, we evaluated the effects of PEMFs both on human adipose mesenchymal stem cells (hASCs) and on primary human osteoblasts (hOBs) by testing gene and protein expression of early bone markers (on hASCs) and the synthesis of late bone-specific proteins (on hOBs) as markers of bone remodeling. Our results indicate that PEMFs seem to exert their action on bone formation, acting on osteogenic precursors (hASCs) and inducing the commitment towards the differentiation pathways, unlike mature and terminally differentiated cells (hOBs), which are known to resist homeostasis perturbation more and seem to be much less responsive than mesenchymal stem cells. Understanding the role of PEMFs on bone regenerative processes provides important details for their clinical application.

## 1. Introduction

Pulsed electromagnetic fields (PEMFs) have been widely used in orthopedic clinical practices to promote bone healing processes [1]. They were approved by the FDA in 1979 for orthopedic treatments such as nonunion of bone, congenital pseudoarthrosis, and failed fusions. Currently, skeletal cells’ responses to PEMFs have been therapeutically evaluated with devices that expose bone cells to electromagnetic fields, promoting extracellular matrix (ECM) synthesis for bone repair [2,3]. Despite its clinical use, cell responses activated by PEMFs in bone tissue are not yet completely known [4]. In literature, it has been observed that PEMFs are able to activate different molecular pathways to promote osteogenesis. Understanding the molecular pathways activated by PEMFs’ exposure provides important details for their clinical application. However, it is still obscure which phase of the bone regenerative process is more responsive to PEMF stimulation.

PEMFs are generated from an alternate current being passed through a coil. They are low-frequency magnetic fields with a specific waveform and amplitude, characterized by a constant variation in the magnetic field amplitude over time. Despite numerous studies about the effect of PEMF stimulations on cell responses, there is no consensus on the optimal parameters (frequency, intensity, and duration) that will promote bone growth and bone healing [5]. Evidence in the literature shows that the most common parameters used in vitro are the following [6]:

- Intensity: ranging from 0.1 mT to 2 mT;
- Frequency: ranging from 15 Hz to 75 Hz;
- Duration: ranging from 8 min to 24 h for many days (from 1 to 28 days).

Most in vitro experiments highlighted a gene expression increase in the main bone markers alkaline phosphatase (*alp*), runt-related transcription factor 2 (*runx2*), osteocalcin (*ocn*), and osteopontin (*opn*); then, the enhancement of alkaline phosphatase (ALP) enzymatic activity and other typical bone matrix proteins was also detected [7].

The bone healing process consists of four distinct overlapping phases: the inflammatory phase (phase 1), in which inflammatory cells migrate to the site of injury; the angio-mesenchymal phase (phase 2), characterized by both vascular remodeling (angiogenesis and neovascularization) and the recruitment of mesenchymal progenitor cells, sometimes referred to as mesenchymal stem cells (MSCs); the bone formation phase (phase 3), in which MSCs previously recruited undergo differentiation to either osteoblasts or chondrocytes, mainly regulated by RUNX2 and SOX9; and finally, the bone remodeling phase (phase 4), in which a balance between osteoclast (OC) and osteoblast (OB) activities is established to maintain bone homeostasis.

**Figure 1:**
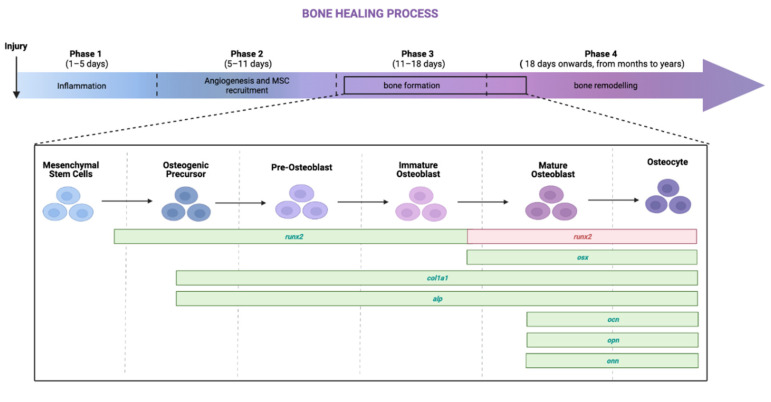
Schematic representation of bone healing process focused on bone formation and part of bone remodeling phases. Runt-related transcription factor (RUNX*2*) is early expressed during bone formation, keeping the osteoblasts in immature state; successively, the simultaneous down-regulation of RUNX2 and the up-regulation of osterix (OSX) allows osteoblasts’ maturation. The activating transcription factor 4 (ATF*4*) regulates osteoblast differentiation, working in cooperation with OSX, promoting bone matrix protein synthesis such as osteocalcin (OCN), which has the ability to bind calcium to modulate the calcium metabolism by mediating its association with hydroxyapatite (HA) [9]; osteopontin (OPN) regulates osteoclast genesis and osteoclast activity, contributing to bone formation and resorption [10,11]; and osteonectin (ONN) is a regulator of the calcium release by binding collagen and HA crystals, thereby influencing the mineralization of collagen during bone formation [12]. Collagen α-1 type I (COL1A1), which can promote osteogenic differentiation and mineralization, and alkaline phosphatase (ALP), which contributes to mineralization [13], at the beginning of phase 3 both are weakly expressed, and then matured osteoblasts increase *col1a1* and *alp* expression. Bone sialoprotein (*ibsp*) is vital in the regulation of osteoblast differentiation and initiation of matrix mineralization in bone tissue, whereas dentin matrix acidic phosphoprotein 1 (DMP1) and sclerostin (SOST) are specific osteocyte markers [14,15]. Created in BioRender.com.

Based on the current literature knowledge, we schematized the four phases of bone healing in Figure 1, highlighting the up- and down-regulation of bone-specific genes. Specifically, in the early phases of phase 3, *runx2*, *alp*, and *col1a1* are expected to increase, whereas once the immature osteoblasts mature, OSX, along with ATF4, is thought to regulate many downstream targets including the genes coding for the bone extracellular matrix proteins [8].

Since it has been observed that PEMFs seem to exert positive effects in phases 2, 3, and 4, while inhibiting the inflammatory phase, the aim of this work was to elucidate if PEMFs exert their action more on osteogenic differentiation of MSCs (phase 3) or on the biosynthetic activities of osteoblasts (phase 4). Thus, we evaluated the effects of PEMFs both on human adipose mesenchymal stem cells (hASCs) and on primary human osteoblasts (hOBs). Specifically, we tested on hASCs the gene and protein expression of early (runx2, alp, and col1a1) bone markers and the synthesis of late bone-specific proteins on (ALP, OPN, OCN, and COL1A1) hOBs as a marker of bone remodeling.

## 2. Results

### 2.1. Proliferation of hASCs

**Figure 2:**
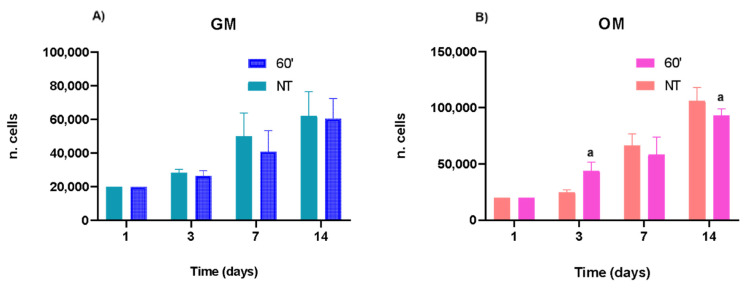
Proliferation of hASCs with and w/o PEMFs. In (**A**), the hASCs kept in GM proliferated during the in vitro culture, showing no cell cycle changes due to PEMF application. In (**B**), the hASCs cultured in OM showed a statistically significant increase in the presence of both osteogenic factors and physical stimuli (PEMFs) versus the untreated cells. All the data reported are expressed as the mean ± S.E.M. of six independent experiments. Before the statistical analysis, the raw data were tested for normality with Kolmogorov–Smirnov normality test, followed by a multiple *t*-test, to check the trend of cell proliferation, and by unpaired *t*-test to verify the differences at each time point (w/o vs. treated cells). The results were evaluated as statistically significant (a) when the *p*-value < 0.05.

The results reported in Figure 2 show that the seeded hASCs, both in GM and OM, were able to replicate along the in vitro culture. Moreover, the results of the multiple *t*-test did not display any change in the proliferative trend of hASCs w/o vs. PEMFs+ both in GM and in OM. However, when checking the differences at each time point, with an unpaired *t*-test we found that the application of PEMFs affects the proliferation of hASCs when treated with osteogenic factors. Specifically, PEMFs caused an increase in cell proliferation at 3 days, a tendency that changes up to 14 days as the osteogenic differentiation proceeds. However, in GM there are no effects, demonstrating that the synergism between biological and physical factors positively contribute to the progression of osteogenic differentiation.

### 2.2. hASCs’ Osteogenic Differentiation—Bone Formation Phase

#### 2.2.1. mRNA Expression

**Figure 3:**
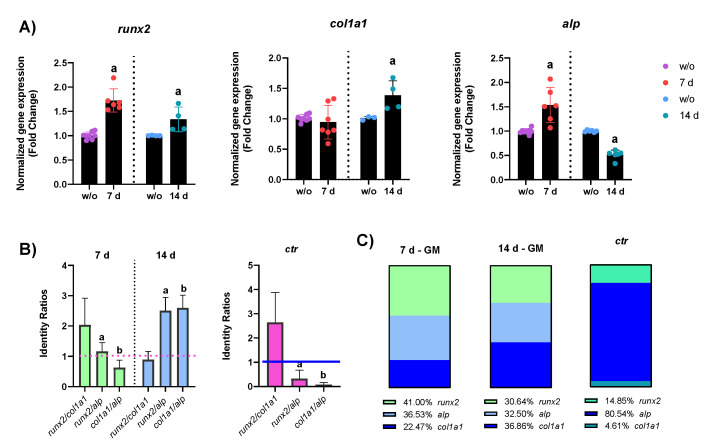

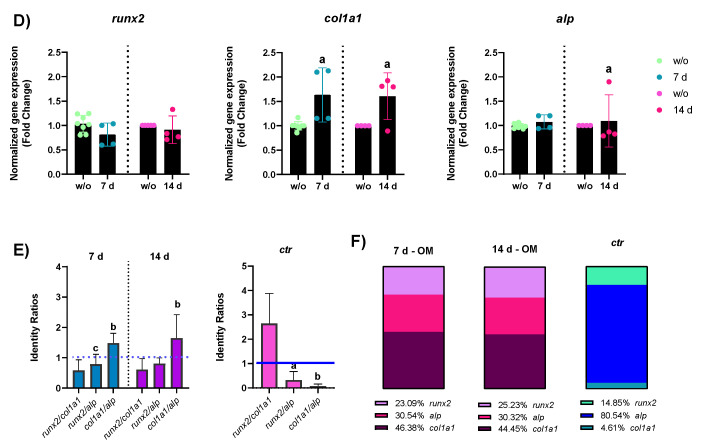
Gene expression results of hASCs cultured both in GM and OM with or w/o the PEMF treatment for 7 and 14 days. The results shown in panels (**A**–**C**) refer to treated and untreated (PEMFs+ and w/o) hASCs kept in GM, whereas the panels (**D**–**F**) show the results of PEMFs+ and w/o hASCs committed towards the osteogenic differentiation (OM). The gene expression results in the bar plots (**A**,**D**) are reported as the mean ± S.E.M. of fold changes (FC). The results in (**B**,**E**) are the identity ratios of treated and untreated hASCs expressed in the plots as the mean ± S.E.M. of FC ratios. Finally, in (**C**,**F**) are reported “part of whole” plots, which highlight the proportion of bone-specific genes (expressed as FC) of treated and untreated hASCs. The bold letters a, b and c in the plots indicate the statistically significant results (*p* < 0.05). In (**A**,**D**), the data were analyzed with an unpaired *t*-test: w/o vs. PEMFs+. In (**B**,**E**), the identity ratios were tested with a One-Way ANOVA followed by Tukey’s test for multiple comparison: a—runx2/col1a1 vs. col1a1/alp; b—runx2/col1a1 vs. col1a1/alp; c—runx2/alp vs. col1a1/alp. All the results reported are the mean ± S.E.M. of six independent experiments, tested for normality and further analyzed with GraphPad Prism 8.4.2.

The mRNA expression of RUNX2, ALP, and COL1A1 was assessed by qRT-PCR in hASCs cultured both in GM and in OM with and without PEMFs. According to the melt curve plot, there was only one peak corresponding to a single amplicon, indicating the specificity of the PCR reaction. The qRT-PCR results of *alp*, *runx2*, and *col1a1* are expressed as fold change versus the expression of untreated hASCs (w/o). After 7 days, the expression levels of both *runx2* and *alp* were statistically higher than those of untreated cells (Figure 3A), whereas at 14 days of treatment, there was a significant increase in the expression of *col1a1*, along with *runx2*, while *alp* resulted down-regulated. We then calculated the identity ratios to define specific gene expression patterns to identify early, intermediate, and late phases of bone formation (phase 3). Then, we compared the gene expression patterns of treated (PEMFs+) hASCs in GM and OM with those of untreated hASCs in OM (ctr). As reported in Figure 3B, the gene expression pattern of ctr and PEMF+ cells at 7 and 14 days were markedly different: in vitro differentiation at 14 days of ctr cells was characterized by the increase in *alp* expression over *runx2* and a low expression of *col1a1*. The application of PEMFs indeed promoted the up-regulation of *col1a1*, even though the process of osteogenic differentiation was at its beginning (early phase), as documented by the up-regulation of *runx2* over *alp*. These discrepancies in the gene expression pattern between the reference (ctr) and the PEMF+ cells (7 and 14 days) are visually represented in Figure 3C. The application of PEMFs to cells cultured with osteogenic factors (OM) caused the up-regulation of *col1a1*. In this case, as reported in Figure 3E, the application of PEMFs along with the treatment with biological factors caused a complete reversal of the gene expression pattern: indeed, while in ctr the relationship between the transcriptional activity of genes was *alp* > *runx2* > *col1a1*, in PEMF+ cells this was *col1a1* > *runx2* > *alp*, suggesting that the synergism between biological factors and physical stimuli was able to promote the progression of osteogenic differentiation.

#### 2.2.2. Protein Expression

**Figure 4:**
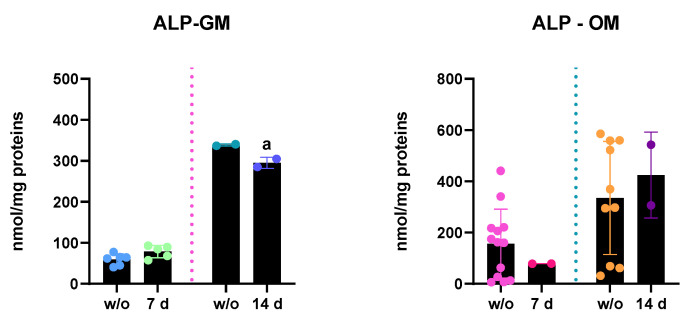
The results shown refer to treated and untreated (+PEMFs and w/o) hASCs kept in GM and OM. The plots show the results of ALP activity, where “a” indicates a statistical significance between w/o and PEMF+ cells, at either 7 or 14 days; in the panel on the right are reported the representative images of ALP activity at 7 days. All the data reported are expressed as the mean ± S.E.M. of six independent experiments. Before the statistical analysis was performed, the raw data were tested for normality with Kolmogorov–Smirnov normality test and further analyzed with unpaired *t*-test on GraphPad Prism 8.4.2. The results were evaluated as statistically significant (a) when the *p*-value < 0.05.

To confirm the functional proteins’ synthesis, ALP activity and RUNX2 expression and localization were evaluated:

- ALP enzymatic activity: ALP activity of hASC lysates was measured after 7 and 14 days of PEMF treatments and results are reported in Figure 4. In accordance with increased ALP mRNA expression, in PEMF+ cells, kept in GM, ALP activity increased after 7 days of treatment, whereas at 14 days of treatment, there was a statistically significant decrease in ALP content versus the w/o cells, in accordance with the reduced gene expression. In OM, instead, after 7 days of PEMF treatment there was a slight decrease in the ALP activity, even if not statistically significant. However, at 14 days ALP activity seemed to increase a little, probably due to the progression of osteogenic differentiation.

- Immunofluorescence staining: Immunofluorescence staining was used to analyze the synthesis of both RUNX2 and COL1A1 proteins.

**Figure 5:**
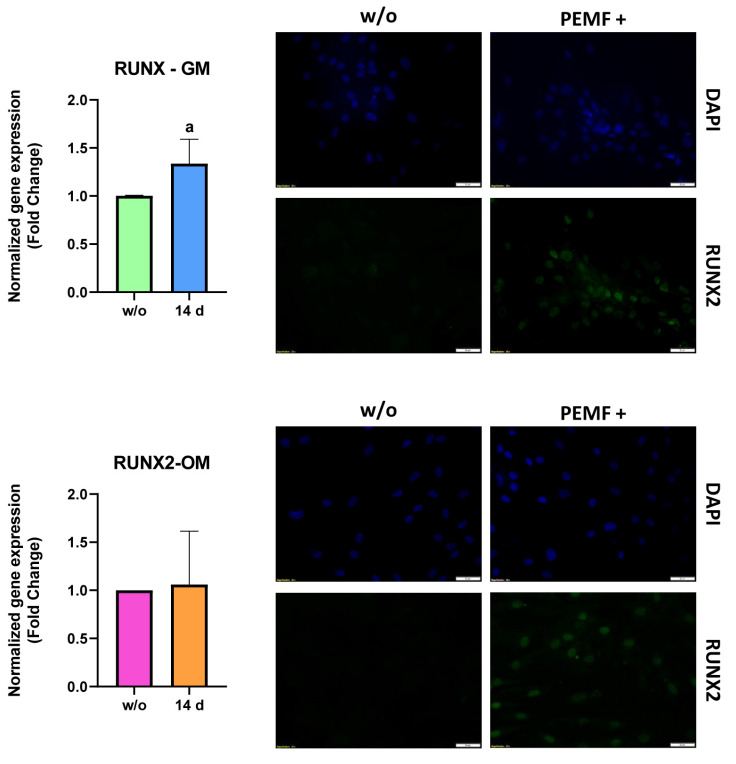
Immunofluorescence images of RUNX2 and gene expression at 14 days culture time. All the data reported are expressed as the mean ± S.E.M. of six independent experiments. Before the statistical analysis was performed, the raw data were tested for normality with Kolmogorov–Smirnov normality test and further analyzed with unpaired *t*-test on GraphPad Prism 8.4.2. The results were evaluated as statistically significant (a) when the *p*-value < 0.05. Scale bar: 50 µm.

RUNX2: As expected, we found that in hASCs w/o either PEMF stimuli or osteogenic factors, there was no signal. Upon the treatment, in accordance with the gene expression results, the mRNA of RUNX2 was actively translated (as documented by the cytoplasmic signal) and imported into the nucleus, promoting the transcription of downstream bone-specific genes, whereas in OM/PEMFs+ cells, there was a milder signal than in GM PEMF+ cells (Figure 5).

**Figure 6:**
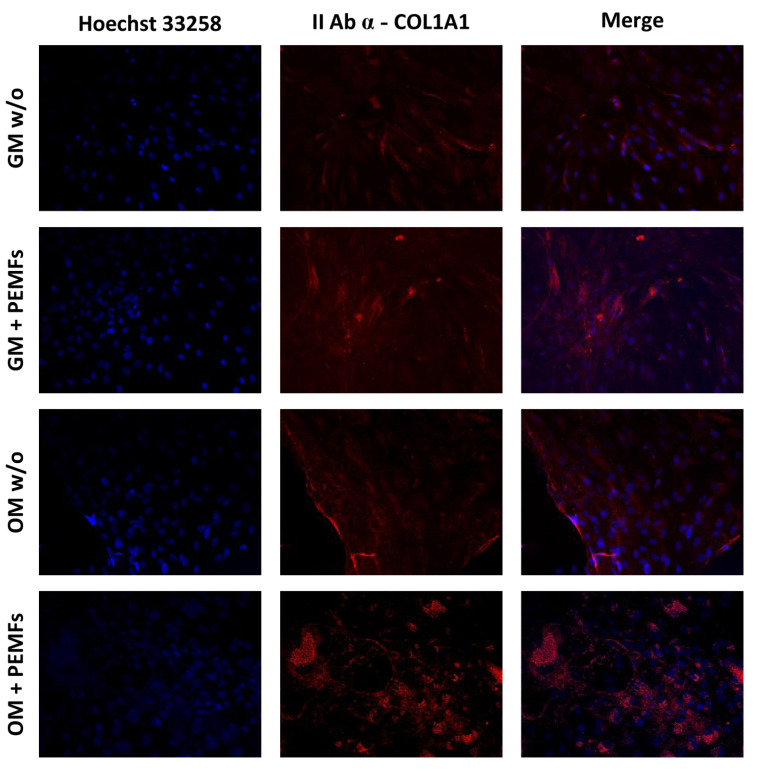
In this panel are reported the IF results of COL1A1 staining. The application of PEMFs induced an increase in collagen I synthesis in hASCs cultured in GM and OM, confirming the gene expression data. Specifically, the application of PEMFs in OM cells induced the major synthesis of collagen I, as demonstrated by the bright spots around the cell nucleus (ER synthesis), supporting the synergistic action of PEMFs and osteogenic factors. Scale bar: 50 µm.

COL1A1: In accordance with the qPCR results, the IF staining reported in Figure 6 showed that the application of PEMFs was able to induce a strong synthesis of collagen type I, in cells cultured in either GM or OM, with the strongest signal in OM + PEMFs cells.

### 2.3. Osteoblast Biosynthetic Activities—Bone Remodeling Phase

**Figure 7:**
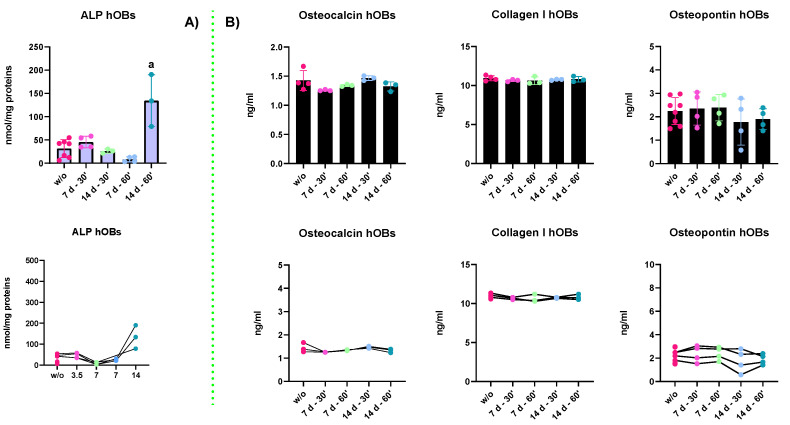
In (**A**) are reported the results of ALP enzymatic activity in hOBs treated with PEMFs for either 30 or 60 min. The only statistically significant difference (a) was detected between 14 d—60′ against all the other experimental groups. In the image below is shown the trend of ALP’s activity depending on the whole treatment’s duration, expressed in hours (h); in (**B**) are reported the results of ELISA assays of OCN, COL1A1, and OPN. As shown in the bar plots, there were no differences in the secreted structural proteins, despite the PEMF treatments. As shown in the plots below, the relative abundance of the proteins or any change in their concentration between the experimental groups was undetectable. All the data reported are expressed as the mean ± S.E.M. of two independent experiments. Before the statistical analysis was performed, the raw data were tested for normality with Kolmogorov–Smirnov normality test and further analyzed with One-Way ANOVA, followed by Tukey’s test for multiple comparisons on GraphPad Prism 8.4.2. The results were evaluated as statistically significant (a) when the *p*-value < 0.05.

Since the aim of the work was to test if the PEMFs interfere with bone formation or remodeling, we tested the biosynthetic activities of osteoblast to verify if PEMF stimuli increase the synthesis and secretion of ECM bone-specific proteins. Moreover, we analyzed the enzymatic activity of ALP to test if these physical stimuli affect the osteoblasts’ mineralization rate:

- ALP enzymatic activity: The results in Figure 7A report the ALP enzymatic activity of hOBs kept in GM for 7 and 14 days and treated for 30 and 60 min. The results indicated that a strong increase in the ALP synthesis and activity was detected upon 14 days of treatment. Indeed, all the other experimental groups displayed a lower ALP content. These results may suggest that hOBs need long-lasting treatments to cause a physiological response. The ALP activity trends depending on the treatment duration are represented in the plot right below. In fact, the relationship between the cellular response and the duration of the stimuli becomes evident. Indeed, the only statistical differences (*p*-value < 0.0001) were found between the 14 d—60′ and all the other groups.

- ELISA assay: ELISA assays were performed to evaluate the secretion of bony ECM proteins. The proteins investigated were collagen type I (COL1A1), osteopontin (OPN), and osteocalcin (OCN). However, the results reported in Figure 7B indicate that irrespective of PEMF treatments, there were no changes in the secretory activities of osteoblasts, an important feature of bone remodeling. Moreover, as shown by the trends in the last line of the figure, there were no changes in the proportion of the secreted proteins in the medium.

## 3. Discussion

PEMF application is widely used to treat a variety of orthopedic clinical conditions, however, the cellular responses activated by these stimuli is still under investigation. In the literature, it has been demonstrated that physical stimulation is increasingly showing extreme importance in the osteogenic differentiation [6]. Studies have demonstrated that appropriate mechanical forces are important for bone metabolism and homeostasis, in particular mechanical forces such as cyclic strain and fluid shear stress [16]. However, other stimuli than solely mechanical treatments emerged as potential alternative strategies to improve bone healing and regeneration, for instance piezoelectricity and ultrasound stimulation. Currently, low-intensity pulsed ultrasound stimulation (LIPUS) is applied as a complementary therapy for the treatment of delayed- and nonunion bones [17]; however, its efficacy is still under investigation. A recently published systematic review [18] critically investigated the studies about the LIPUS effects and concluded that it might be helpful for the treatment of the above-listed conditions; however, they may fail if atrophy or bone inertness occur. However, predicting the clinical outcome of these alternative treatments is challenging, due to the lack of proper randomized clinical trials and double-blinded studies. Another physical stimulus that has demonstrated to modulate bone regeneration is the piezoelectricity [19,20,21]. Indeed, several in vitro and in vivo studies reported that the use of piezoelectric biomaterials was able to induce both the osteogenic differentiation of stem cells as well as to promote the vascularization of the implanted biomaterial [22]. However, despite several physical stimuli that seem to be able to help bone regeneration, an accurate comparative study is missing. Even though we are aware of the difficulties in comparing alternative strategies when standard guidelines are missing, we suggest and propose that a deep comparison of different physical stimuli on bone healing would help the scientific community to focus on the more promising alternative therapy. In this work, we focused on the molecular effects of pulsed electromagnetic fields on bone regeneration. Indeed, unlike other physical stimuli, extensive in vitro data were available [23], thus offering the ground for a deeper investigation, to understand in which phase of bone healing PEMFs had the major effects. This comprehension will help the many efforts of worldwide clinicians to trace guidelines for the clinical application of PEMFs.

As described previously (Figure 1), the bone healing process consists of four distinct but overlapping phases. Since it has been observed that PEMFs seem to exert positive effects in steps 2–4 of bone regeneration, while inhibiting the inflammatory phase, the aim of this work was to elucidate if PEMFs exert their action more on osteogenic differentiation of MSCs (phase 3) or on the biosynthetic activities of osteoblasts (phase 4). Then, we tested the osteogenic effects of PEMFs by analyzing both the gene expression patterns and the protein expression of all experimental groups: not treated (ctr), 7 d GM PEMFs+, 14 d GM PEMFs+, 7 d OM PEMFs+, and 14 d OM PEMFs+.

The qRT-PCR results demonstrated that the application of PEMFs in hASCs kept in GM was able to trigger the osteogenic differentiation, as documented by the up-regulation of *runx2*, *alp*, and *col1a1* (Figure 3). Specifically, the up-regulation of *runx2* and *alp* occurred earlier, as soon as 7 days of PEMF treatment, whereas *col1a1* displayed a “late” up-regulation. These results are in line with the current scientific literature knowledge, which consider ALP an early marker of osteogenic differentiation. However, the role of ALP at the beginning of osteogenesis is unknown: while its role in bone mineralization is clear, its function during the initiation of osteogenic differentiation is still obscure [24,25]. As a phosphatase, it is likely that it might have a role in mediating the activation of signaling cascades to induce the osteogenic differentiation. The calculus of identity ratio and the relative abundance of expressed genes (Figure 3B,C) indicates that *runx2* has the strongest transcriptional activity [26]. For these reasons, the gene expression profile was indicative of the early phase of bone formation. In contrast, 14 days of treatment induces an increase in the *col1a1* and *runx2* gene expression, causing *alp* down-regulation. The slight down-regulation of *alp* might be linked to the polyhedric functions of this protein: it is well recognized that both kinases and phosphatases are subjected to a quick and sharp transcriptional and post-translational regulation [27]. Due to the increase in *col1a1* expression and a slight decrease in *alp*, we postulated that the PEMFs + hASCs entered the intermediate phase of bone regeneration. The PEMFs + hASCs kept in OM reported a strong increase in *col1a1* expression already at 7 days of treatment, as the commitment towards the osteogenic process probably started earlier, due to the administration of dexamethasone, ascorbic acid, and β-glycerophosphate. When looking at Figure 3E, the identity ratios of PEMFs + hASCs displayed a reversal trend of the expression pattern with respect to ctr. While untreated cells were characterized by a strong expression of *alp*, the PEMF application caused the up-regulation of *col1a1*, favoring the progression towards the end of the intermediate phase. To conclude, the gene expression analysis revealed that, in the presence of osteogenic factors, PEMFs seem able to accelerate the progression of osteogenic differentiation, promoting the shift from the early to the end of the intermediate phase of bone formation.

Moreover, we tested the expression and localization of ALP, COL1A1, and RUNX2 to confirm the qRT-PCR data. The results reported in Figure 4 confirmed ALP mRNA expression data: at 7 days of treatment, the hASCs kept in GM displayed a significant increase in the ALP activity, which is further down-regulated (14 days of treatment). Even in this case, the mild down-regulation of ALP (the same occurring in mRNA expression) right after the osteogenic commitment might be indicative of osteogenic progression towards the intermediate phase of bone formation. The treated GM hASCs showed the presence of RUNX2 both in the cytoplasm and in the nucleus, indicating that the protein was actively translated (mRNA up-regulation) and translocated into the nuclear compartment to execute its function (Figure 5). When the hASCs were treated with both physical stimuli (PEMFs) and biological factors (dexamethasone, ascorbic acid, and β-glycerophosphate), the gene expression of *runx2* of PEMFs+ and w/o was steady. However, the protein expression was different: the PEMFs’ application induced active translation and translocation into the nucleus, suggesting that there might be a post-transcriptional control of RUNX2 mRNA, regulating its translation rates. Finally, the results reported in Figure 6 indicated that the PEMFs’ treatment induced a stronger synthesis of collagen I in both GM- and OM-cultured cells, confirming the gene expression results. Specifically, it appears that the GM PEMFs+ and OM w/o cells had similar COL1A1 content, suggesting that the application of PEMFs alone can induce ECM synthesis and deposition.

As the aim of our work was to evaluate if PEMFs’ application had effects on bone remodeling (phase 4), we tested both the biosynthetic capabilities of hOBs and their mineralization rate. The biosynthetic and secretory capabilities were evaluated on supernatants of osteoblasts by ELISA assays. However, the results demonstrated that all treatments (7 d, 14 d, 30 min, or 60 min) were not able to modify the synthesis or the release of extracellular bone-specific proteins, such as osteopontin, osteocalcin, and collagen type I (Figure 6). The mineralization rates were assessed by ALP enzymatic assay: the results indicated that only long-lasting treatments (14 days) induced a physiological response of osteoblasts, whereas shorter treatments, even though with same physical features (intensity, shape, and frequency), did not induce any perturbation of the ALP synthesis. These results overall indicate that PEMFs seem to exert their action on phase 3 of bone formation, acting on osteogenic precursors. Indeed, it is possible that the plasticity of stem cells (hASCs were considered in our work) made them more sensitive and responsive to external stimuli, thus inducing the commitment towards the differentiation pathways [28]. In addition, shorter treatments (7 days) were able to induce quick responses both in gene and protein expression, whereas mature and terminally differentiated cells (mature hOBs in our work) are known to resist homeostasis perturbation more. In fact, only long-lasting treatments were able to induce a massive increase in ALP synthesis, known to have a role in bone mineralization. However, the ALP enzymatic assay only gives us information about protein concentration. For these reasons, a specific staining assay is required to determine and quantify the presence of hydroxyapatite depositions and to analyze the mineralization status of PEMFs + hOBs.

Moreover, these results may be strictly due to the protocols adopted for this work. Indeed, we performed several preliminary qPCR tests to understand which could be the best time exposure to achieve a biological response. Based on these tests, we decided to treat the cells 60 min per day. If these parameters were able to induce changes in the hASCs, they might be insufficient to detect changes in the biosynthetic capabilities of osteoblasts. For these reasons, we could not exclude the effects of PEMFs on bone remodeling. This is an extremely interesting result, since it suggests that there might exist a real dose–response effect, which would help the resolution of a variety of clinical conditions interfering with different phases of bone healing.

**Figure 8:**
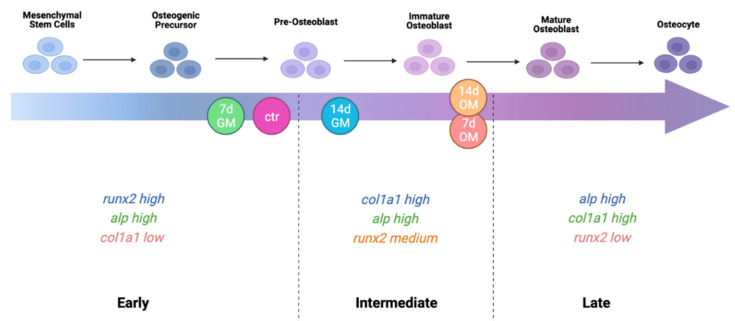
Schematic figure that represents our proposal of the dynamic gene expression patterns occurring during phase 3 of bone formation. This image has been conceptualized based on the current literature knowledge and our experimental data. Moreover, the differentiation patterns reported here refer to the differentiation of hASCs, thus they might be different for other stem cell populations. Based on the calculus of identity ratios (FC ratios, Figure 3), we recognized the relative expression of bone-specific genes. We propose a division of the bone formation phase in early, intermediate, and late phases. As *runx2* is the master regulator of osteogenic differentiation known to be activated at the beginning of bone formation, its overexpression with respect to *alp* and *col1a1* may be a marker of the early bone formation phase. As the differentiation proceeds, *alp* expression is expected to gradually increase up to the late phase of bone formation. The same occurs for the expression of *col1a1*; however, it is generally considered a late marker of differentiation along with all the other structural extracellular matrix proteins [4,32].

Based on these results and the current findings in the literature, we also proposed a scheme of the bone formation (phase 3) gene expression patterns (Figure 8). As during the osteogenic differentiation process the relative abundance of the expression levels of *runx2*, *alp*, and *col1a1* are expected to change, we calculated and classified the identity ratios of each experimental group in early, intermediate, and late phases of bone formation. During the osteogenic commitment (early phase of bone formation), *runx2* is the most up-regulated gene, which functions by promoting the transcriptional activation of downstream bone-specific genes, such *alp* and *col1a1.* However, the intermediate phase of bone formation, regulating the differentiation up to the immature osteoblast, is characterized by a milder expression of *alp* and an up-regulation of *col1a1*, the most abundant bony ECM protein. In this phase, *runx2* is still expressed, as it regulates the progression of differentiation up to the immature osteoblasts, even if the proportion of its downstream targets increases gradually (ratio *runx2*/*col1a1* and *runx2*/*alp* < 1.0). At the end, in the late phase there is a strong increase in both *col1a1* and *alp* expression, linked to biosynthetic activities of osteoblasts and mineralization processes.

However, despite the encouraging results, this study needs further investigation: specifically, more bone-specific genes and transcription factors should be analyzed to have a broader and a clearer view about the progression of osteogenic differentiation. Indeed, the bone markers used in this study may have a non-linear gene expression trend, making the data analysis and interpretation difficult. For instance, even though the involvement of ALP in the osteogenic differentiation is well documented, its gene expression pattern during the bone formation is still obscure [29], as it is often considered both as an early and late marker of bone formation. Moreover, several studies reported an increased collagen I deposition upon the PEMFs’ stimulation [30,31]; however, this increase does not necessarily accompany the osteogenic differentiation, as the hASCs themselves express a great amount of collagen I. However, even though the *col1a1* gene expression cannot be considered a tight marker of osteogenic differentiation, its augmented expression and deposition have, in any case, beneficial roles for tissue repair and regeneration. Finally, the up-regulation of *runx2* in treated cells is a clear hint of stem cells’ differentiation; however, how far the osteogenic differentiation goes with the synergistic action between OM and PEMFs’ application cannot be accurately predicted because of the lack of well-known late markers such as osx, onn, ibsp, ocn, and opn.

Thus, considering both the results and the limits of our study, we suggest and encourage the scientific community to focus mainly on two points.

First, the role of early, intermediate, and late markers of bone formation must be clarified, to build up a model of gene expression changes that may help the comprehension of the molecular mechanisms occurring during both tissue regeneration and embryo development.

Second, the roles PEMFs may play in fracture healing and bone regeneration and in the broader context of regenerative medicine must be detailed better.

## 4. Materials and Methods

**Figure 9:**
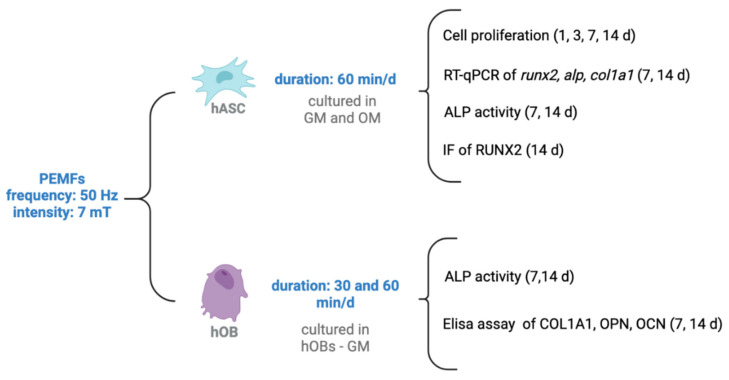
Schematic representation of the experimental design used to evaluate osteogenic differentiation (on hASCs) and bone remodeling (on hOBs). Created in BioRender.com.

To verify if PEMFs’ application induces or alters the osteogenic differentiation of hASCs (phase 3 of bone regeneration), these cells were tested for the gene (*runx2*, *alp*, and *col1a1*) and protein expression (RUNX2 and ALP) of bone-specific markers. The biosynthetic activities of osteoblasts (ALP activity, OPN, OCN, and COL1A1 secretion), a marker of bone remodeling (phase 4 of bone regeneration), were tested with ALP and ELISA assays, as schematized in Figure 9.

### 4.1. Types of Cells

- Human adipose-derived stem cells (hASCs)

Subcutaneous adipose tissue was obtained from healthy donors during hip replacement surgery. The study was conducted in accordance with the 1975 Declaration of Helsinki; informed consent was obtained from all patients before surgery and the protocol was approved by the Ethics Committee of San Matteo Foundation, Research and Care Institute, Pavia, Italy (P-20190023312, 9 April 2019). The samples preserved in sterile conditions were transported to the laboratory for processing. In brief, the finely minced tissue was incubated in digestion buffer (0.01% collagenase type II in DMEM F12-HAM medium) for 1 h at 37 °C in a shaking water bath [33]. At the end of the incubation time, the collagenase was neutralized, and the suspension filtered and centrifuged at 1200 rpm for 10 min at 4 °C. The pellet was washed twice with PBS, treated with lysis solution, and finally suspended in growth medium (GM, DMEM F12-HAM supplemented with 10% FBS, 100 U/mL penicillin, 100 μg/mL streptomycin, and 0.25 µg/mL amphotericin). The hASCs were cultured in GM up to 95% confluence in a humidified atmosphere of 95% air with 5% CO_2_ at 37 °C. The adherent cells were trypsinized with Trypsin EDTA, and 5000 hASCs/cm^2^ tissue culture plate were seeded in a new flask [34]. These passages were repeated three times. At the third passage, the hASCs were positive for the mesenchymal stem cell markers CD73, CD90, and CD105 and negative for the hematopoietic cell markers CD34 and CD45, according to the analysis performed by flow cytometer (Navios Beckman Coulter). Data were acquired, displayed, and elaborated by Kaluza 1.2 software package (Beckman Coulter Indianapolis, IN, USA). The positive cells were counted and compared with the signal of corresponding immunoglobulin isotypes [6].

- Human Osteoblasts (hOBs)

The primary human osteoblasts, purchased from PromoCell, were cultured in osteoblast growth medium (C-27001, PromoCell, Heidelberg, Germany) in a humidified atmosphere of 95% air with 5% CO_2_ at 37 °C. At 95% confluence, the hOBs were detached with Trypsin EDTA and further expanded until their experimental processing.

### 4.2. Cell Seeding and PEMF Treatment

At the third passage, both the hASCs and hOBs were trypsinized and seeded in monolayer at a density of 6000 cells/cm^2^. Then, the hASCs were cultured in GM and in osteogenic medium (OM, DMEM F12-HAM containing 15% FBS, 10 mM β-glycerophosphate, 100 nM dexamethasone, 0.05 mM ascorbic acid, antibiotics, and amphotericin). Half of the plates were treated with PEMFs using Osteoplus^®^ tissue biostimulator (provided by Tesla Medical S.r.l., Napoli, Italy) at a frequency of 50 Hz and intensity of 7 mT, for 60 min per day. The parameters were chosen by preliminary qPCR tests performed in our lab and by a consistent literature review [3,4]. The untreated hASCs were referred to as “control”, whereas the osteoblasts were treated with the same parameters (50 Hz and 7 mT) for 30 and 60 min, to exclude the hypothesis that PEMFs may have a stronger effect even at lower exposure duration on already differentiated cells. After 7 and 14 days of treatment, all the cells were sacrificed to carry on the experiments, as explained below.

### 4.3. Proliferation Assay (WST)

Cell proliferation was evaluated with the Quick Cell Proliferation Colorimetric Assay Kit (Abcam, Waltham, MA, USA, #K301) according to the manufacturer’s instructions. The assay is based on the degradation of the tetrazolium salt WST-1 to formazan by cellular mitochondrial dehydrogenase. The dye amount generated by activity of dehydrogenase is directly proportional to the number of living cells. The formazan dye produced by viable cells can be quantified by measuring the absorbance of the dye solution at 450 nm. hASCs cultured in GM and OM were incubated with 10% WST working solution for 2 h at 37 °C (95% air with 5% CO_2_), and then the absorbance was read using a spectrophotometer. To determine the number of cells, a calibration curve was built.

### 4.4. RNA Isolation, Reverse Transcription, and Quantitative Real-Time PCR (qRT-PCR)

After 7 and 14 days from the cell seeding, RNA was extracted with QIAzol Lysis Reagent. The total RNA isolated was reverse-transcribed into cDNA using random hexamers and M-MLV Reverse Transcriptase, according to Laforenza et al., 2010 [7]. Quantitative real-time PCR (qRT-PCR) was performed in triplicate using 2 µL cDNA obtained as above, using specific primers from Qiagen: *alp* (QT00012957), *runx2* (QT00020517), and *col1a1* (QT00037793). Quantifast-SYBR Green PCR Kit (Qiagen) was used according to the manufacturer’s instruction and qRT-PCR was performed using Rotor Gene 6000 (Corbett). Cycling conditions: initial denaturation at 95 °C for 5 min; 40 cycles of denaturation at 95 °C for 30 s; annealing at 60 °C for 30 s; and elongation at 72 °C for 40 s. Melting curves were generated to identify the melting temperatures of specific products after the PCR run. The qRT-PCR reactions were normalized against the expression of the housekeeping *β2m* gene (beta-2 micro-globulin, QT00088935, Qiagen, Hilden, Germany). The gene expression’s results were expressed as fold change versus the expression of untreated cells.

### 4.5. Alkaline Phosphatase (ALP) Activity

To confirm osteogenic differentiation, ALP activity was evaluated [35]. The culture medium was removed, and the cells were trypsinized with Trypsin EDTA, rinsed in PBS, and incubated with 5 mM p-nitrophenyl-phosphate in 50 mM glycine, 1 mM MgSO_4_, 1 mM ZnSO_4_, and pH 10.5 at 37 °C for 30 min. The absorbance of p-nitrophenol formed was read at 410 nm. The ALP activity was expressed as 1 nmol p-nitrophenol/mg proteins. The proteins’ concentration was evaluated with Coomassie Blue Staining and the absorbance was read using a spectrophotometer at 595 nm.

### 4.6. Immunofluorescence (IF)

After 14 days of culture, the cells grown on slides were fixed with paraformaldehyde at 4% (PFA 4%) in PBS1x for 30 min. To facilitate the entry of primary antibody into the cell nucleus, the fixed cells were permeabilized with TRITON-X 0.4%. Then, the slides were washed trice with PBS 1X and incubated at room temperature (RT) with PBS-BSA 3% to block aspecific sites. Subsequently, cells were incubated overnight at 4 °C with the diluted (1:200) primary antibody against either RUNX2 (Boster Immunoleader M00442) or COL1A1 (1:50) (PA1-26204, Thermo Fisher Scientific, Waltham, MA, USA). Thereafter, the slides were washed trice with PBS 1X and incubated with secondary antibody diluted 1:1000 (ab150081, abcam and A-21207, Thermo Fisher Scientific) for 30 min at RT. Then, the nuclei were counterstained with Hoechst 33,258 at RT for 5 min [36]. Finally, the slides were mounted with an anti-fading mounting solution and kept at 4 °C until their visualization with the fluorescence microscope Nikon Eclipse 80i (Nikon, Tokyo, Japan).

### 4.7. ELISA Assays

The ECM protein secretion into the medium was quantified by ELISA assays (Cloud-Clone Corp., Katy, TX, USA). The exhausted medium was collected from the hOBs’ flasks. Then, the medium was centrifuged to discard the cell debris and further processed following the manufacturer’s instructions. The purchased kits were targeting collagen type I (SEA571Hu 96Tests), osteopontin (SEA899Hu 96 tests), and osteocalcin (SEA471Hu 96 tests).

### 4.8. Statistics

All the data reported were expressed as the mean ± standard error (S.E.M.). Before the statistical testing, the raw data were tested for normality with Kolmogorov–Smirnov normality test, followed by either One-Way ANOVA or *t*-tests (multiple or unpaired). The differences were considered statistically significant when the *p*-value < 0.05. The statistical analyses were performed with a dedicated software: GraphPad Prism 8.4.2. (GraphPad Software, La Jolla, CA, USA, www.graphpad.com, accessed on 23 July 2022).

## 5. Conclusions

To conclude, our results indicate that the PEMFs’ application seemed to exert major effects on phase 3 of bone formation: the qRT-PCR results showed changes in the expression pattern that might be indicative of osteogenic differentiation progression towards the end of the intermediate phase. Human osteoblasts (referred to phase 4) seemed to be less responsive than mesenchymal stem cells (referred to phase 3) when treated with these physical parameters. Moreover, only long-lasting treatments were able to induce a physiological response, associated with mineralization activities. In addition, ECM protein secretion was not affected at all by the application of PEMFs. Although these are encouraging results, it seems that PEMFs acted on specific molecular axes, rather than on the overall cellular physiology. From a clinical point of view, it would be interesting to test PEMFs’ effects on patients with pathologies (such as pseudoarthrosis, nonunion or late union, or osteoarthritis [37]) to establish the possible therapeutic dose–response. In fact, it seems that PEMFs can modulate the biological processes in different pathological contexts, probably depending on the settled parameters. For these reasons, it is necessary to deepen the real dose–response relationship to understand the plausible PEMFs’ applications in clinical practice: understanding which bone regenerative phases are more prone to be positively affected by PEMFs allows and helps the scientific community to establish international guidelines for the temporal application of PEMFs according to the in vivo regenerative timescale.

## Data Availability

Not applicable.

## Abbreviations

ALP/*alp*	alkaline phosphatase protein/gene
ATF4/*atf4*	activating transcription factor 4 protein/gene
B2M/*β2m*	beta-2-microglobulin protein/gene
COL1A1/*col1a1*	collagen α-1 type I protein/gene
ctr	control
dmp1	dentin matrix acidic phosphoprotein 1
ECM	extracellular matrix
FBS	fetal bovine serum
GM	growth medium
hASCs	human adipose mesenchymal stem cells
hOBs	primary human osteoblasts
BSPII/*ibsp*	bone sialoprotein II protein/gene
IF	immunofluorescence
MSCs	mesenchymal stem cells
OBs	osteoblasts
OCN/*ocn*	osteocalcin protein/gene
OCs	osteoclasts
OM	osteogenic medium
ONN/*onn*	osteonectin protein/gene
OPN/*opn*	osteopontin protein/gene
OSX/*osx*	osterix protein/gene
PBS	phosphate buffered saline
PEMFs	pulsed electromagnetic fields
PFA	paraformaldehyde
qRT-PCR	quantitative real-time polymerase chain reaction
RT	room temperature
RUNX2/*runx2*	runt-related transcription factor 2 protein/gene
SOX9/*sox9*	transcription factor 9 protein/gene
WST-1	water-soluble tetrazolium 1
